# Hepatic FoxO1 Acetylation Is Involved in Oleanolic Acid-Induced Memory of Glycemic Control: Novel Findings from Study 2

**DOI:** 10.1371/journal.pone.0107231

**Published:** 2014-09-15

**Authors:** Xiu Zhou, Xiao-Yi Zeng, Hao Wang, Songpei Li, Eunjung Jo, Charlie C. L. Xue, Minjia Tan, Juan C. Molero, Ji-Ming Ye

**Affiliations:** 1 Molecular Pharmacology for Diabetes, Health Innovations Research Institute and School of Health Sciences, Royal Melbourne Institute of Technology, Melbourne, Victoria, Australia; 2 State Key Laboratory of Drug Research, Shanghai Institute of Materia Medica, Chinese Academy of Sciences, Shanghai, China; Northeast Ohio Medical University, United States of America

## Abstract

Our recent study (referred as Study 1) showed that the triterpenoid oleanolic acid (OA) was able to produce a sustained correction of hyperglycemia beyond treatment period in type 2 diabetes (T2D) mice with liver as a responsible site. To follow up the previous observations, the present study (referred as Study 2) investigated the possible role of acetylation of FoxO1 and associated events in this therapeutic memory by characterizing the pathways regulating the acetylation status during and post-OA treatments. OA treatment (100 mg/kg/day for 4 weeks, during OA treatment) reduced hyperglycemia in T2D mice by ∼87% and this effect was largely (∼70%) maintained even 4 weeks after the cessation of OA administration (post-OA treatment). During OA treatment, the acetylation and phosphorylation of FoxO1 were markedly increased (1.5 to 2.5-fold) while G6Pase expression was suppressed by ∼80%. Consistent with this, OA treatment reversed pyruvate intolerance in high-fat fed mice. Histone acetyltransferase 1 (HAT1) content was increased (>50%) and histone deacetylases (HDACs) 4 and 5 (not HDAC1) were reduced by 30–50%. The OA-induced changes in FoxO1, G6Pase, HAT1 and HDACs persisted during the post-OA treatment period when the increased phosphorylation of AMPK, SIRT1 content and reduced liver triglyceride had subsided. These results confirmed the ability of OA to control hyperglycemia far beyond treatment period in T2D mice. Most importantly, in the present study we demonstrated acetylation of FoxO1 in the liver is involved in OA-induced memory for the control of hyperglycemia. Our novel findings suggest that acetylation of the key regulatory proteins of hepatic gluconeogenesis is a plausible mechanism by the triterpenoid to achieve a sustained glycemic control for T2D.

## Introduction

Type 2 diabetes (T2D) is a major disease with serious consequences [1]. As hyperglycemia is a major cause of the organ damages, it is essential to control the hyperglycemia effectively to prevent its complications [2]. Recent studies including ours have indicated that triterpenoid compounds may emerge as potential anti-diabetic drugs with distinct therapeutic properties [3], [4]. In humans, triterpenoids have demonstrated promising therapeutic effects for diabetic complications such as nephropathy [5] where prolonged hyperglycemia is a major culprit [6].

Several mechanisms have been proposed to be involved in the anti-diabetic effects of triterpenoids. For example, in both L6 myotubes and 3T3-L1 adipocytes, triterpenoids have been found to stimulate the translocation of GLUT4 from cytosol to plasma membrane via the AMP-activated protein kinase (AMPK) pathway [4]. AMPK has been reported as a major cellular target of several anti-diabetic small molecules, namely metformin [7], thiazolidinedione (pioglitazone and rosiglitazone) [8] and berberine (BBR) [9]. Interestingly, our recent studies showed that these triterpenoids may activate AMPK by a mechanism entirely different from these anti-diabetic small molecules [10]. Additionally, triterpenoids has been found to suppress mitochondrial ROS, inflammation and glucose-6-phosphatase (G6Pase) expression in the liver of *db/db* mice [11]. These studies described above indicate that triterpenoids might be a valuable source for the discovery of new efficacious anti-diabetic drugs. Moreover, as reviewed recently, the mechanism for the anti-diabetic properties of triterpenoids is unlikely to be confined to the simultaneous presence of AMPK activation [3], particularly after the removal of the treatment.

Oleanolic acid (OA) is a pentacyclic triterpenoid abundantly available in the plant kingdom and it has been used for the treatment of lung cancer [12]. We recently observed that OA was able to substantially reduce hyperglycemia in a mouse model of T2D generated by high-fat diet (HF) plus low doses of streptozotocin (STZ) injections and this effect was sustained for at least 4 weeks after the cessation of its administration (referred as Study 1) [13]. With the use of glucose tracers, we identified the suppression of hepatic glucose production as a source of the sustained normalization of blood glucose in Study 1 [13]. However, the mechanism involved remained to be investigated. Acetylation of key regulators has been suggested to involve in metabolic memory to normalize hyperglycemia [14], [15]. To follow up the previous observation in Study 1, the present study (referred as Study 2) investigated the possible role of acetylation of FoxO1 and associated events in this therapeutic memory by comparing their responses during and post-OA treatments. To ensure the consistency of the treatments, Study 2 was performed under similar designs and experimental conditions as Study 1 but in different cohorts of mice.

## Materials and Methods

### Animal experiments

Ten-week-old male C57BL/6J mice (Animal Resources Centre, Perth, Australia) were housed at 22±1°C on a 12-hour light/dark cycle with free access to water and food. After one week of acclimatization, mice were fed *ad libitum* a standard lab chow (CH) diet (12% calories from fat, 65% calories from carbohydrate and 23% calories from protein) or a HF diet (45% calories from fat, 35% calories from carbohydrates and 20% calories from protein) for 10 weeks to generate insulin resistance as described previously [16]. HF-fed mice were then injected with vehicle (saline) or low doses of STZ (40 mg/kg/day, to block the compensatory secretion of insulin) for 5 consecutive days to generate a T2D mouse model (T2D mice) [17], [18]. Once hyperglycemia (fasting blood glucose >14 mM) was developed, one group of T2D mice received OA (100 mg/kg/day) in the HF diet for 4 weeks (during OA treatment, T2D-OA) before tissue collection; another group of T2D mice received OA (100 mg/kg/day) in the HF diet for 2 weeks and then fed an OA-free HF diet for another 4 weeks (post-OA treatment, T2D-OA) before tissues collection, while the rest of the mice remained on either the CH or HF diet. Body weight, food intake and blood glucose were monitored weekly. Mice were sacrificed by cervical dislocation. All experiments were carried out with the approval of the Animal Ethics Committees of the Royal Melbourne Institute of Technology University (Project #1012) in accordance with the guidelines of the National Health and Medical Research Council of Australia.

### Measurement of oxygen consumption in isolated mitochondria

Mitochondria were isolated as described previously [9]. Briefly, rat livers were homogenized in medium containing 250 mM sucrose, 10 mM Tris-HCl, 1 mM EGTA, and 1% fatty acid free BSA, pH 7.4. Mitochondrial respiration was measured at 37°C with a Clark-type oxygen electrode (Strathkelvin Instruments, Scotland) in a respiration medium containing 225 mM mannitol, 75 mM sucrose, 10 mM Tris-HCl, 10 mM KH_2_PO_4_, 10 mM KCl, 0.8 mM MgCl_2_, 0.1 mM EDTA and 0.3% fatty acid-free BSA, pH 7.0. The effects of different compounds were determined in the presence of excess ADP (2.4 mM), using substrate combinations targeting either Complex I (5 mM pyruvate plus 2 mM malate) or Complex II (10 mM succinate plus 4 µM rotenone) of the respiratory chain.

### Assessment of blood glucose, blood insulin and liver triglyceride

Blood glucose was measured using a glucometer (AccuCheck II, Roche, Australia) while insulin was determined by radioimmunoassay (Linco/Millpore, USA). At the end of the study, mice were killed by cervical dislocation and the relevant tissue samples were immediately freeze-clamped. Triglyceride levels in plasma and liver were determined by a Peridochrom triglyceride GPO-PAP kit (Roche Diagnostics, USA) [19].

### Assessment of glucose and pyruvate tolerance

Intraperitoneal glucose tolerance tests (ipGTT, 1.0 g glucose/kg body weight) were performed after 5–7 hours fasting after 2 weeks of OA administration and 2 weeks after the cessation of OA administration. Blood glucose levels were measured at 0, 15, 30, 60 and 90 min and plasma insulin levels were sampled at 0, 15, 30 and 60 min. The effect of OA on hepatic glucose production from gluconeogenesis was examined in another cohort of mice. Briefly, mice fed a HF diet for 8 weeks were treated with OA at the same dose (100 mg/kg/day) in the HF diet for the last 2 weeks. The pyruvate tolerance test (PTT) was performed with an intraperitoneal injection of sodium pyruvate (2.0 g/kg body weight in 1xPBS) after overnight fasting. Blood glucose levels were measured at 0, 15, 30, 60 and 90 min.

### Western blot analysis

Freeze-clamped liver tissues were homogenized in ice-cold RIPA lysis buffer at pH 7.5 supplemented with protease and phosphatase inhibitor cocktails. Protein levels in tissue homogenates were determined using the bi-cinchonnic acid method (Bio-Rad Laboratories Inc., USA). Liver lysates containing equal amount of proteins were resolved by SDS-PAGE. Activation of key insulin signaling proteins and levels of lipogenic and gluconeogenic enzymes were examined by immunoblotting using specific antibodies. Forkhead box protein O1 (FoxO1), p-FoxO1^Ser256^, HDAC4, p-HDAC4^Ser632^, HDAC5, p-HDAC5^Ser498^, HDAC1, AMPK, p-AMPK^Thr172^, acetyl-CoA carboxylase (ACC), p-ACC^Ser79^, fatty acid synthase (FAS), stearoyl-CoA desaturase 1 (SCD1), Akt, p-Akt^Ser473^, glycogen synthase kinase 3β (GSK3β) and p-GSK3β^Ser9^ were obtained from Cell Signaling (USA). Acetyl-FoxO1^lys259, 262 and 271^ and sterol regulatory element-binding protein 1c (SREBP-1c) were purchased from Santa Cruz (USA). HAT1 and sirtuin 1 (SIRT1) were from Abcam and Millipore (USA) respectively. Quantitative densitometry analysis was performed using Image Lab software (Bio-Rad Laboratories Inc., USA).

### Quantitative real-time PCR

Total RNA was extracted from liver tissues with TRIZOL (Invitrogen, USA) according to manufacturer's instructions. Reverse transcription was performed with 0.125 µg of RNA using a high capacity cDNA reverse transcription kit (Applied Biosystems, USA). Real time PCR was conducted using IQ SYBR Green Supermix (Bio-Rad Laboratories Inc., USA). The gene expression from each sample was analyzed in duplicates and normalized against the housekeeper *18S*. The primer sequences (Genework, Australia) (5′ to 3′) were as follows: *18S*, CGCCGCTAGAGGTGAAATTCT (sense) and CGAACCTCCGACTTTCGTTCT (antisense); phosphoenolpyruvate carboxykinase (PEPCK), CCACAGCTGCTGCAGAACA (sense) and GAAGGGTCGCATGGCAAA (antisense); G6Pase, AACGCCTTCTATGTCCTCTTTC (sense) and GTTGCTGTAGTAGTCGGTGTCC (antisense). All reactions were performed on the iQ™ 5 Real Time PCR Detection System (Bio-Rad Laboratories Inc., USA).

### Statistical Analysis

Data are presented as mean ± SE. One-way analysis of variance (ANOVA) was used for comparison of relevant groups. When significant variations were found, the Dunnett's multiple comparisons test was applied. Differences at p≤0.05 were considered to be statistically significant.

## Results

### Sustained correction of hyperglycemia induced by OA treatment

We first confirmed the effects of OA treatment on glucose and lipid metabolism in a model of T2D mice [13]. HF-feeding and low-dose of STZ injections induced typical characteristics of the late stage of T2D including hyperglycemia (>2 fold), hypertriglyceridemia (∼80%) and hepatic steatosis (2.2-fold) (all p<0.01 vs. CH-fed mice, Table 1 left panel). OA treatment (T2D-OA) normalized hyperglycemia and hypertriglyceridemia in T2D mice and significantly reduced hepatic steatosis (by 33%) (all p<0.01 vs. T2D mice, Table 1 left panel). The T2D-OA group also displayed improved glucose tolerance (30%, p<0.01, Table 1 left panel) and slightly greater plasma insulin availability during ipGTT. While body weight was reduced by ∼9% (p<0.01, Table 1 left panel), there was no significant change in caloric intake or plasma insulin levels in OA-treated T2D mice. 4 weeks after the termination of OA, these mice still maintained a normalized glucose level and improved glucose tolerance (both p<0.01 vs. T2D mice, Table 1 right panel) despite a full regain of body weight and hepatic steatosis (p>0.05 vs. T2D mice, Table 1 right panel).

**Table 1:** Metabolic responses during and post OA administration in T2D mice induced by HF-STZ.

	During OA treatment			Post-OA treatment		
	CH	T2D	T2D-OA	CH	T2D	T2D-OA
Body weight (g)	29.8±0.5	27.7±0.3	25.3±1.4**†	31.9±0.8	30.2±0.7	29.3±0.7*
Caloric intake (kcal/mouse/day)	13.6±0.7	12.2±0.6	10.9±0.7	10.9±0.5	12.2±1.2	14.1±1.3
Blood glucose (mM)	9.6±0.2	21.3±0.4**	11.1±1.2††	10.0±0.5	26.3±1.3**	14.8±2.3††
ipGTT AUC (mM ×90 min)	1442±38	2902±71**	2045±171**††	1494±71	2609±131**	1838±98**††
Blood insulin (pg/ml)	361±37	306±45	240±21**	367±29	301±63	212±12**
Blood insulin (5–60 min, pg/ml)	417±37	181±13**	241±28**†	441±36	191±18**	280±37**
Plasma triglyceride (mM)	1.1±0.1	2.1±0.2**	1.3±0.2††	1.6±0.1	2.0±0.4*	1.8±0.1
Liver triglyceride (µmol/g)	7.6±0.9	16.8±0.9**	11.3±0.6*††	12.9±0.9	18.9±0.9**	24.9±2.3**

**During treatment**: 4-week administration of oleanolic acid (OA, 100 mg/kg/day in HF diet). Parameters were measured within the last 2 weeks of treatment. **Post-treatment**: 2-week administration of OA (100 mg/kg/day in HF diet) followed by feeding with an OA-free HF diet for 4 weeks. Parameters were measured within the last 2 weeks of the post-treatment period. CH, chow fed mice (normal control); T2D, HF-fed mice with STZ injections (untreated T2D); T2D-OA, T2D with OA treatment. AUC, area under the curve of blood glucose levels during an ipGTT. Data are shown as means ± SE. * p<0.05, ** p<0.01 vs. corresponding CH; † p<0.05, †† p<0.01 vs. corresponding T2D. n = 7–8 mice per group.

### Changes in FoxO1 activity and the expression of its downstream gluconeogenic genes

**Figure 1:**
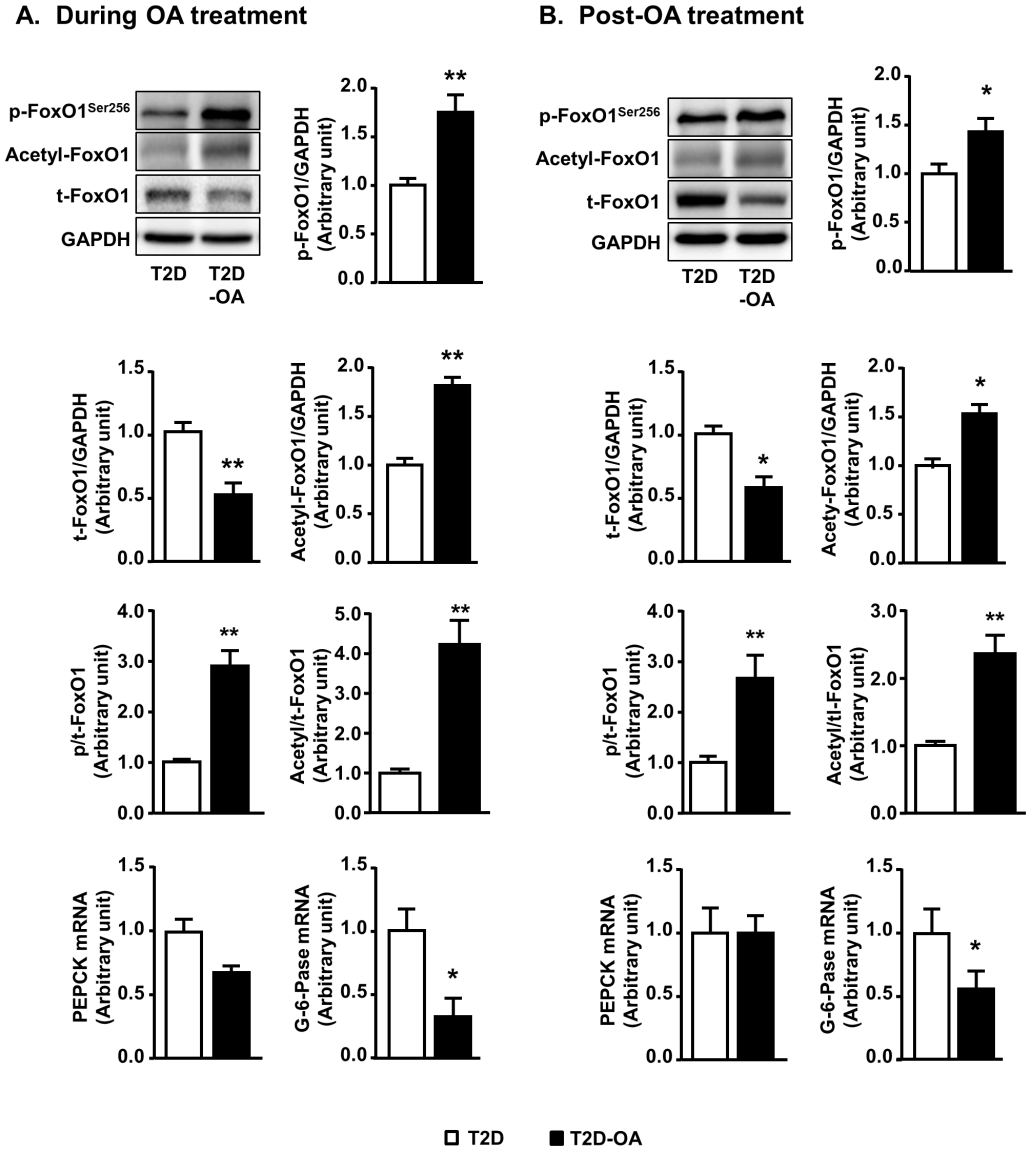
Changes in phosphorylation and acetylation of FoxO1 and the gene expression of PEPCK and G6Pase in the liver. Mice liver samples were freeze-clamped after 4 weeks of OA administration (During OA treatment) (A) or 4 weeks after the cessation of OA administration (Post-OA treatment) (B). Liver lysates from mice were immunoblotted with phosphorylated (p-), acetylated (ac-), total (t-) FoxO1 and quantified for statistical analysis. A separate aliquot of liver sample was extracted for quantitative analysis of the gene expression of PEPCK and G6Pase. Data are mean ± SE. *p<0.05, **p<0.01 vs. T2D mice. n = 5–8 mice per group.

Liver plays an essential role to maintain glucose homeostasis particularly during fasted states by gluconeogenesis. FoxO1, one of the key regulators, mediates the expression of key genes (G6Pase and PEPCK) in the hepatic gluconeogenic pathway. We firstly examined the changes of FoxO1 and its downstream genes. T2D mice showed decreased phosphorylation of FoxO1 (1.00±0.07 vs.1.43±0.10 of CH mice, p<0.05, n = 5–8) and increased total content of FoxO1 (1.00±0.13 vs. 0.75±0.12 of CH mice, n = 5–8) compared to non-diabetic CH mice. The phosphorylation of FoxO1 in the liver was augmented by 1.7-fold and 1.4-fold during and post-OA treatment, respectively (Fig. 1A, B). In line with the increased phosphorylation of FoxO1, its total content was reduced by 50% both during (p<0.01, Fig 1A) and after the treatment (p<0.05, Fig 1B). These results were consistent with our previous observations in Study 1 [13].

In addition, acetylation of FoxO1 has been reported to reduce the expression of key enzymes in gluconeogenesis [14], [15], [20]. However, the possible role of acetylation was not investigated in Study 1 [13]. In the present study, we reasoned whether or not the sustained glycemic control initiated by OA might result from the long-term acetylation of FoxO1. In T2D mice, the acetylation of FoxO1 was markedly reduced compared to non-diabetic CH mice (1.00±0.07 vs. 1.48±0.08 of CH mice, p<0.05, n = 5–8). OA treatment restored the acetylation of FoxO1 at the specific residues of lysine 259, 262 and 271 (1.7-fold, p<0.01 vs. T2D, Fig 1A). The removal of OA did not alter the increased acetylation of FoxO1 established by the treatment (Fig 1B).

Along with this, the mRNA expression of G6Pase, which is a rate-limiting regulator for gluconeogenesis, was also found to be up-regulated in the T2D mice (1.00±0.17 vs. 0.72±0.15 of CH mice, during OA treatment; 1.00±0.27 vs. 0.59±0.21 of CH mice, post-OA treatment, all n = 5–8). Consistent with the changes in FoxO1, the gene expression of G6Pase was down-regulated in the T2D mice during OA treatment (∼80%, Fig 1A), which was maintained in post-OA treatment (∼50%, both p<0.05 vs. T2D mice, Fig. 1B). Although there was a trend of the down-regulation of PEPCK during OA treatment, which is another rate-limiting regulator for gluconeogenesis, this trend was not sustained after the cessation of OA treatment.

### Effect of OA on hepatic glucose production from pyruvate

**Figure 2:**
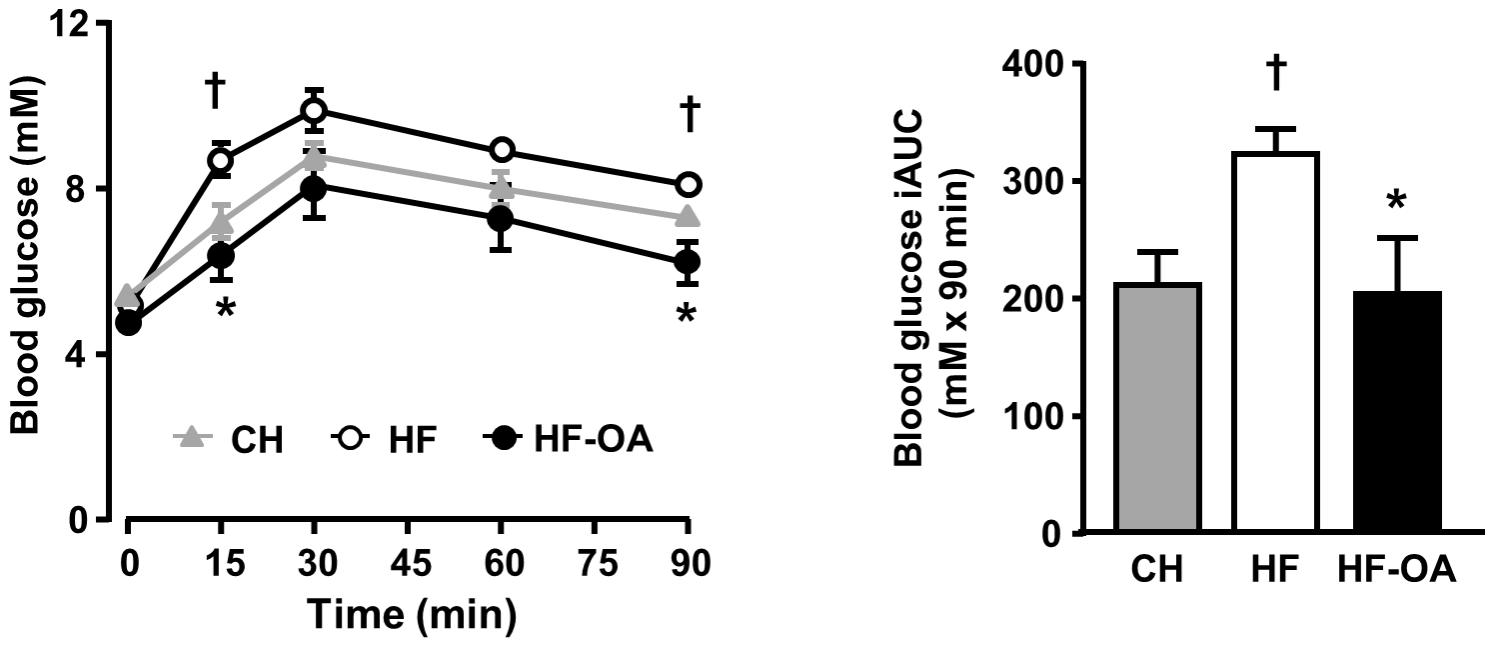
Effect of OA treatment on pyruvate tolerance test in high-fat fed mice. Mice were fed with HF diet for 8 weeks and treated with OA (100 mg/kg/day) in the HF diet for the last 2 weeks. The pyruvate tolerance test was performed with an intraperitoneal injection of sodium pyruvate (2.0 g/kg body weight in 1xPBS) after overnight fasting. Left panel, blood glucose levels were measured at 0, 15, 30, 60 and 90 min; Right panel, incremental area under the curve (iAUC). CH: chow fed mice; HF: high-fat fed mice; HF-OA: high-fat fed mice treated with OA (100 mg/kg/day in diet for 2 weeks). Data are mean ± SE. *p<0.05, vs. T2D mice. †p<0.05 vs. CH mice. n = 6–8 mice per group.

To determine whether OA affects hepatic glucose production *in vivo*, we next examined the effect of OA treatment on pyruvate tolerance in HF-fed mice. As shown in Fig. 2, HF feeding significantly induced pyruvate intolerance compared with CH-fed mice, as indicated by a 50% increase in the incremental area under the curve (iAUC) of blood glucose (322.6±20.9 vs. 210.8±28.0 mM of CH mice, p<0.05, n = 6–8). OA dramatically suppressed the increased glucose production in HF-fed mice by ∼40% (p<0.05).

### Changes in histone acetyltransferase and deacetylases in the liver

Recent studies indicate that acetylation of key transcription factors can maintain an induced phenotype even the trigger is no longer present [15], [21]. Protein acetylation is regulated by histone acetyltransferases (HATs) and deacetylases (HDACs) [22]. We found that the content of Class IIa HDACs, namely HDAC4 (1.00±0.11 vs. 0.70±0.03 of CH mice, p<0.05, n = 5–8) and HDAC5 (1.00±0.25 vs. 0.57±0.08 of CH, p<0.05, n = 5–8) were elevated in the liver of T2D mice whereas the expression of HAT1 was not altered in T2D mice (1.00±0.08 vs. 0.80±0.04 of CH mice, p>0.05, n = 5–8).

**Figure 3:**
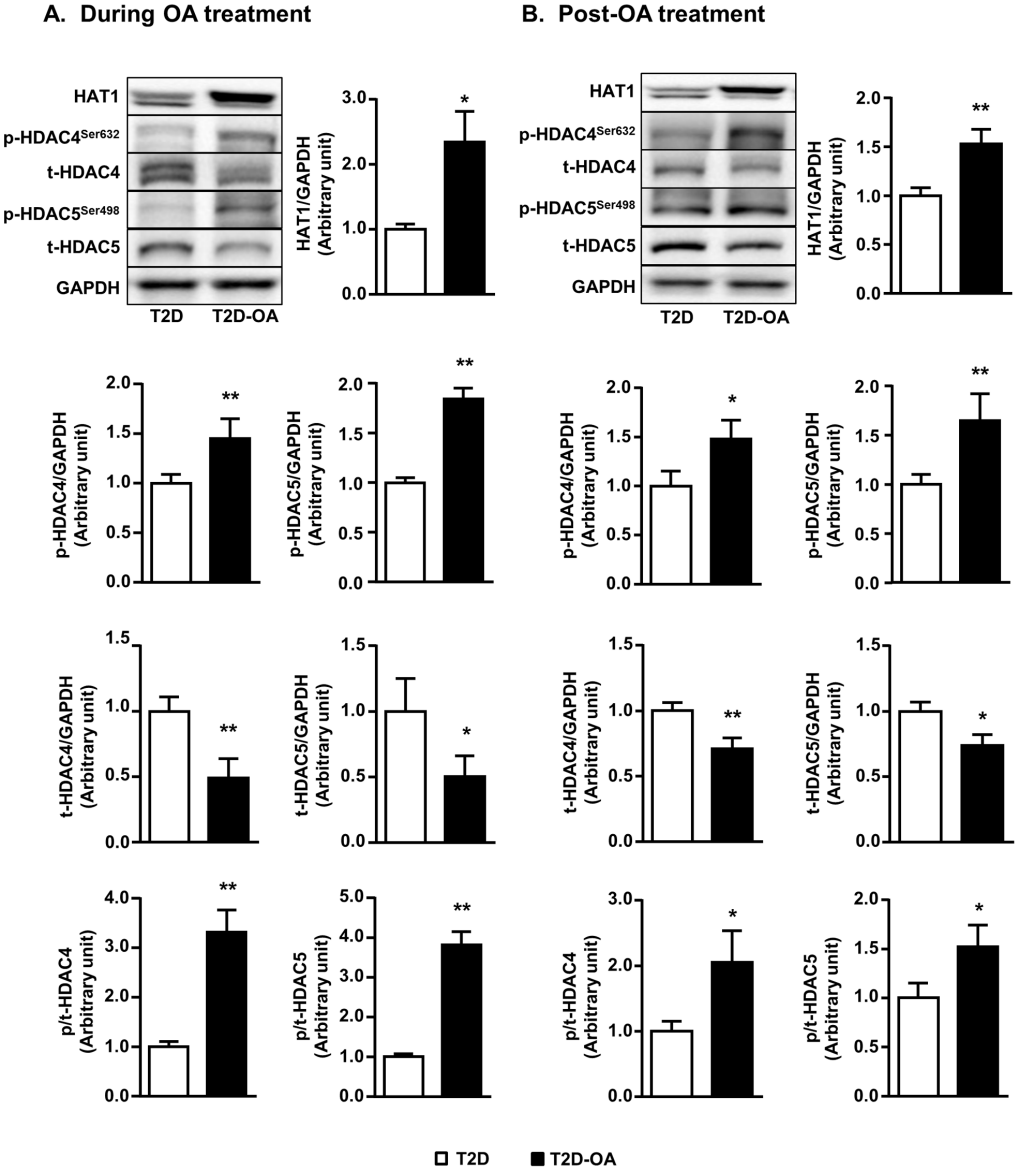
Changes in histone acetyl-transferase 1 and Class IIa histone deacetylases in the liver. Mice liver samples were freeze-clamped after 4 weeks of OA administration (During OA treatment) (A) or 4 weeks after the cessation of OA administration (Post-OA treatment) (B). Liver lysates from mice were immunoblotted with HAT1, phosphorylated (p-) and total (t-) HDAC 4 and 5 (Class IIa HDACs) and quantified for statistical analysis. Data are mean ± SE. *p<0.05, **p<0.01 vs. T2D mice. n = 5–8 mice per group.

**Figure 4:**
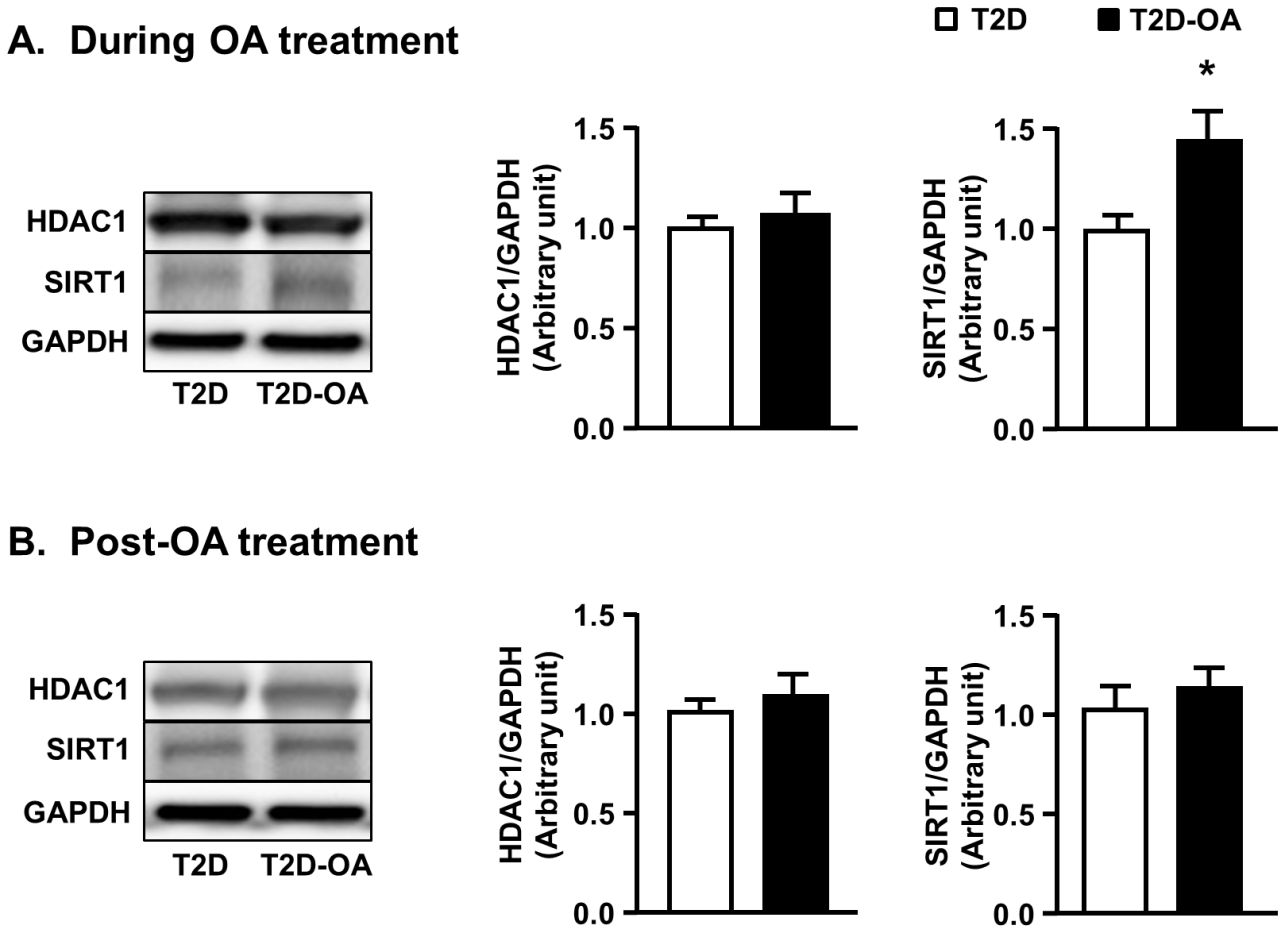
Changes in Class I HDAC and SIRT1 in the liver. Mice liver samples were freeze-clamped after 4 weeks of OA administration (During OA treatment) (A) or 4 weeks after the cessation of OA administration (Post-OA treatment) (B). Liver lysates from mice were immunoblotted with HDAC1 (Class I HDAC) and SIRT1 (Class III HDAC) and quantified for statistical analysis. Data are mean ± SE. *p<0.05, vs. T2D mice. n = 5–8 mice per group.

As shown in Fig. 3A, OA treatment induced an 80% increase in HAT1 content in T2D mice (p<0.05 vs. T2D mice) while the expression of HDAC4 and HDAC5 were markedly reduced (50%, p<0.05). The reduced expression of these HDACs was associated with increases in their phosphorylation (1.5–3-folds, p<0.01), which is indicative of protein deactivation [22]. Removal of OA did not alter the pattern of changes in HAT1 content and the acetylation of HDAC4 and HDAC5 (Fig. 3B). In comparison, there was no significant change in the protein levels of HDAC1 (a Class I HDAC) in response to OA treatment (Fig. 4A, B). The expression of SIRT1 was reduced in the liver of T2D mice compared to non-diabetic CH mice (1.00±0.05 vs. 1.48±0.10 of CH mice, p<0.05, n = 5–8) and OA treatment induced a 50% increase (p<0.05 vs. T2D mice, Fig. 4A) in the expression of SIRT1 (a Class III HDAC). However, this increase was not maintained after the cessation of OA treatment (Fig. 4B).

### Changes in the AMPK pathway and its downstream key lipogenic enzymes in the liver

**Figure 5:**
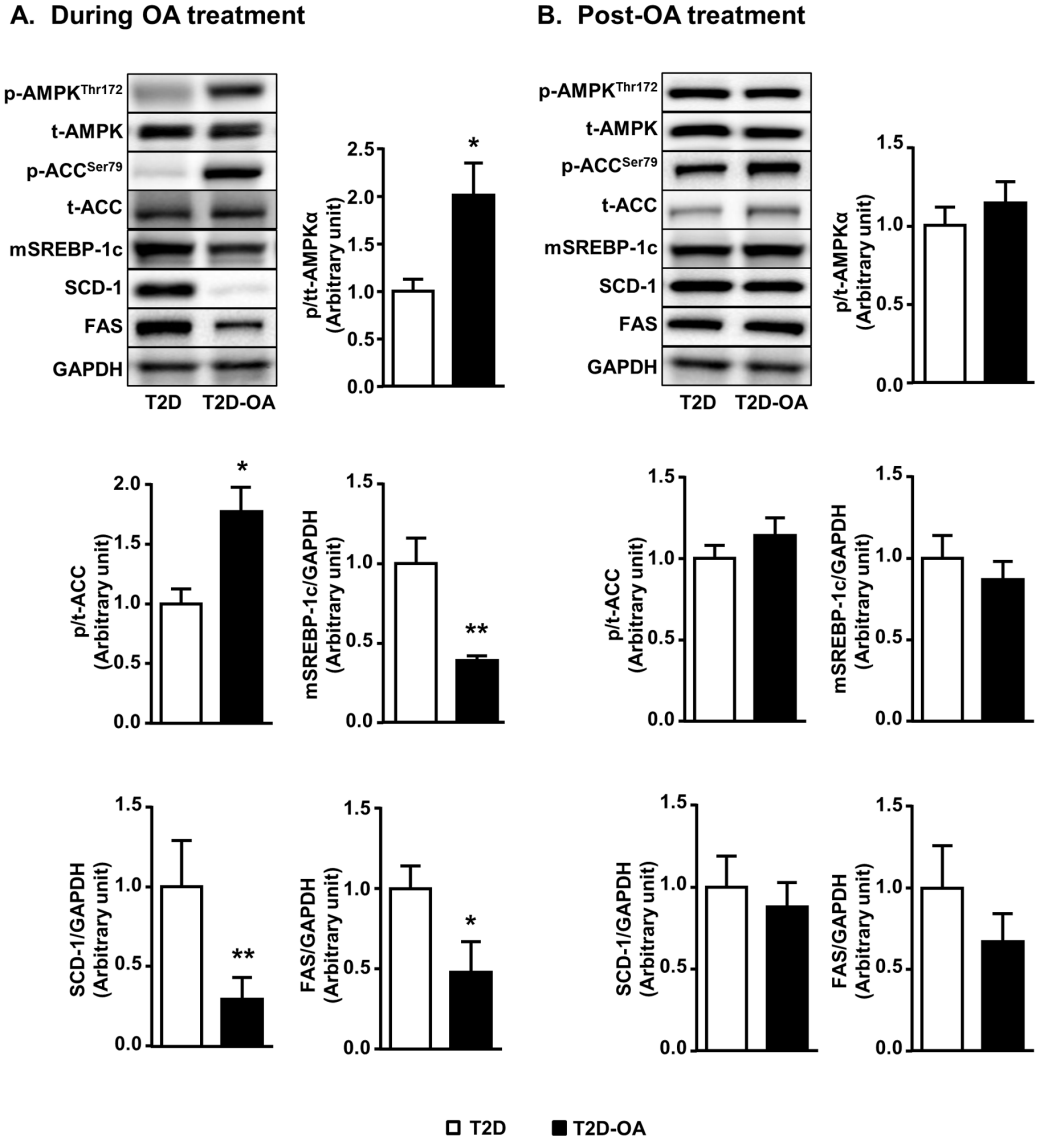
Changes in the AMPK pathway and its downstream key lipogenic enzymes in the liver. Mice liver samples were freeze-clamped after 4 weeks of OA administration (During OA treatment) (A) or 4 weeks after the cessation of OA administration (Post-OA treatment) (B). Liver lysates from mice were immunoblotted with phosphorylated (p-) or total (t-) AMPK, and ACC, mature form of SREBP-1c (mSREBP-1c), SCD-1, FAS and quantified for statistical analysis. Data are mean ± SE. *p<0.05 vs. T2D. n = 5–8 mice per group.

Triterpenoids have been shown to activate AMPK [4] and activated AMPK can phosphorylate/acetylate FoxO1 to suppress hepatic gluconeogenesis [23], [24]. However, the possible role of AMPK and its downstream targets in the metabolic response following OA treatment was not examined in Study 1 [13]. We found in the present study that phosphorylation of AMPK was reduced in T2D mice (1.00±0.13 vs. 2.08±0.24 of CH-fed mice, p<0.01, n = 5–8). OA administration restored the phosphorylation of AMPK (2-fold, p<0.05, Fig. 5A) and its downstream effector ACC (1.7-fold, p<0.05, Fig. 5A). The expression of the mature form of SREBP-1c (mSREBP-1c), SCD-1 and FAS were increased in T2D mice compared to non-diabetic mice (1.00±0.16 vs. 0.80±0.06 of CH mice; 1.00±0.29 vs. 0.51±0.19 of CH mice, p<0.05; 1.00±0.14 vs. 0.88±0.15 of CH mice, all n = 5–8, respectively). Consistent with the effect on AMPK and ACC, OA treatment significantly reduced the levels of mature SREBP-1c (mSREBP-1c by 58%), FAS (by 50%) and SCD-1 (by 61%) (all p<0.05 vs. T2D mice, Fig. 5A). However, the effect of OA on the phosphorylation of AMPK and ACC were not maintained after cessation of OA (Fig. 5B). Coincided with the subsided activation of AMPK, the protein levels of mSREBP1c, FAS and SCD-1 were returned to similar levels as the T2D group (Fig. 5B).

**Figure 6:**
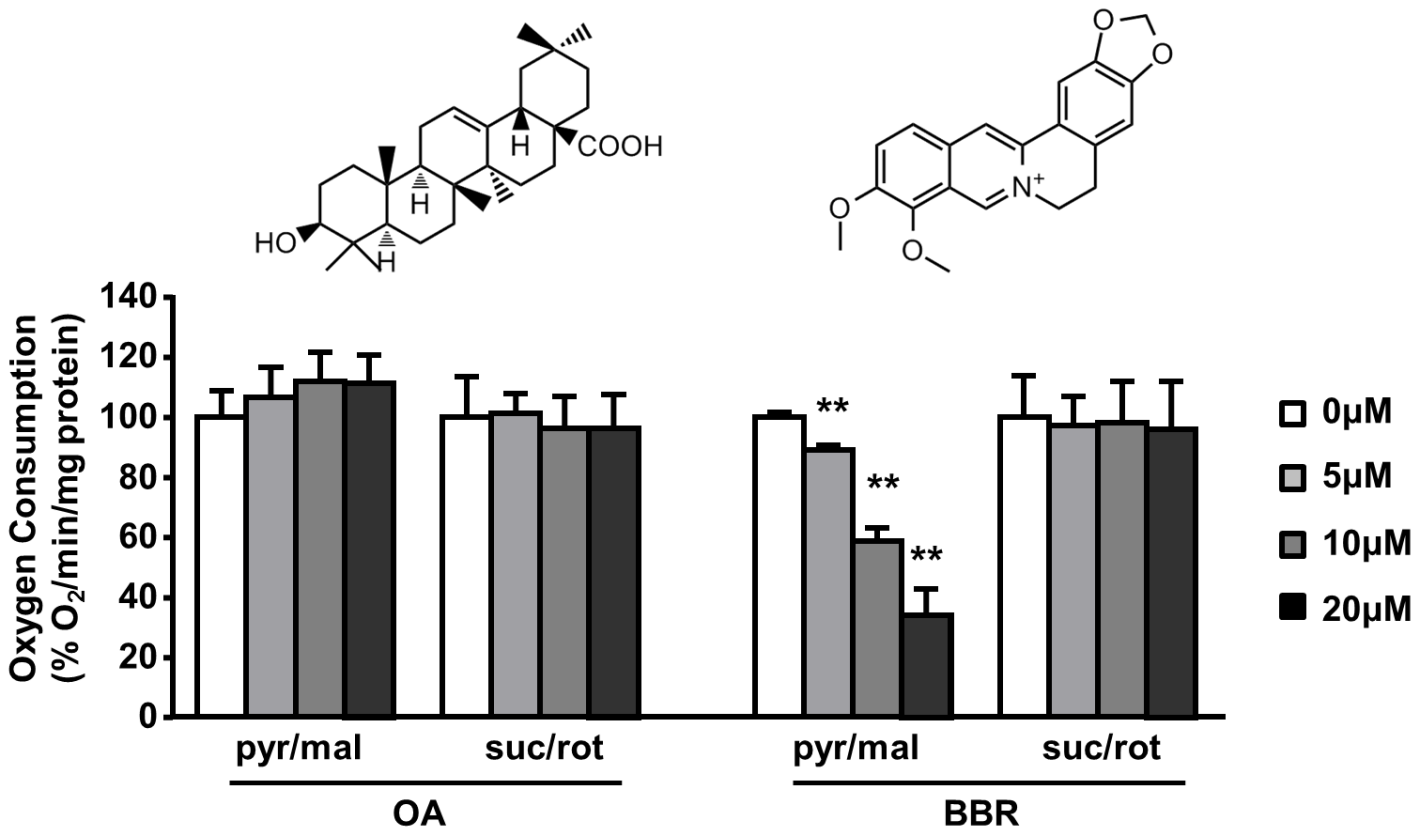
Effect of OA on the respiration of isolated mitochondria from the liver. Mitochondria from rat liver were isolated and the effects of OA/BBR were determined in the presence of excess ADP (2.4 mM), using substrate combinations targeting either Complex I (5 mM pyruvate plus 2 mM malate) or Complex II (10 mM succinate plus 4 µM rotenone) of the respiratory chain. Data are mean ± SE. *p<0.05, **p<0.01 vs. vehicle control (0 µM). n = 3 per group.

Metformin, rosiglitazone and BBR for their anti-diabetic effects and these agents also activate AMPK via inhibiting mitochondrial respiration [9], [25], [26]. We therefore examined whether OA may also inhibit the respiration of mitochondria isolated from the rat liver using BBR as a positive control. As shown in Fig. 6, OA treatment had no effects on mitochondrial respiration regardless of substrates supplied for Complex I (pyruvate plus malate) or Complex II (succinate with the presence of rotenone). Similar results were observed in mitochondria isolated from the skeletal muscle (data not shown). In contrast to OA, BBR attenuated mitochondrial respiration in a dose-dependent manner via inhibition of Complex I.

### Changes in phosphorylation of Akt and GSK3β in the liver

**Figure 7:**
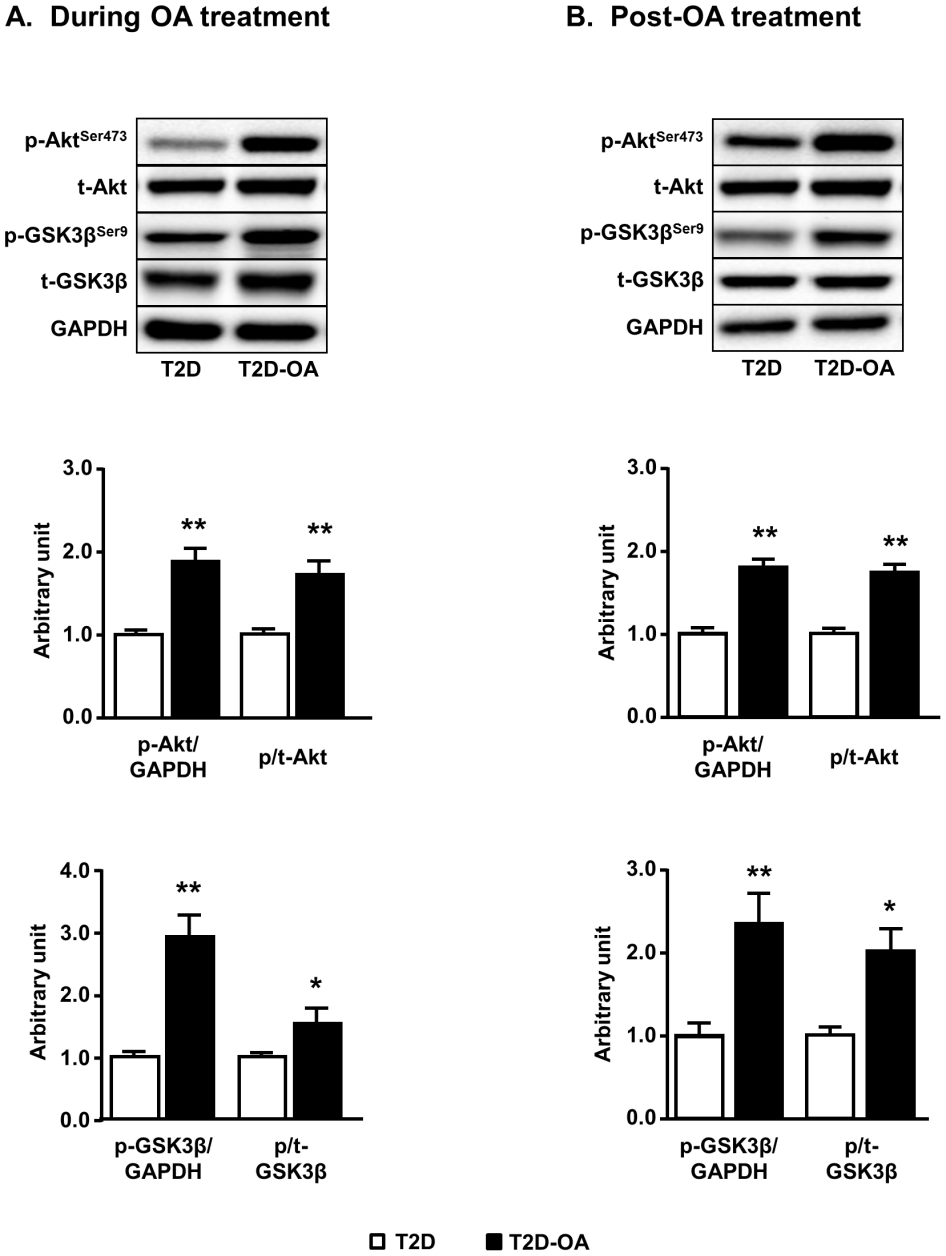
Changes in the phosphorylation of Akt and GSK3β in the liver. Mice liver samples were freeze-clamped after 4 weeks of OA administration (During OA treatment) (A) or 4 weeks after the cessation of OA administration (Post-OA treatment) (B). Liver lysates from mice were immunoblotted with phosphorylated (p-) or total (t-) Akt and GSK3β, and quantified for statistical analysis. Data are mean ± SE. *p<0.05, **p<0.01 vs. T2D mice. n = 5–8 mice per group.

As FoxO1-induced suppression of hepatic gluconeogenesis is also under the regulation of Akt [27], we examined the phosphorylation of Akt as well as its downstream target GSK3β as a proxy of their enzymatic activity. T2D mice exhibited reduced phosphorylation of Akt (1.00±0.06 vs. 1.97±0.09 of CH mice, p<0.01, n = 5–8) and its downstream effector, GSK3β (1.00±0.08 vs. 1.60±0.21 of CH mice, p<0.05, n = 5–8) throughout the course of experiment. As shown in Fig. 7, the phosphorylation of Akt was increased by approximately 2 folds during OA treatment (Fig. 7A) and this increase was sustained after the cessation of OA treatment (Fig. 7B). A similar pattern of changes in the phosphorylation of its downstream target GSK3β was observed both during and post-OA treatments.

## Discussion

Oleanolic acid (OA), a pentacyclic triterpenoid abundantly available in the plant kingdom [12], has been shown to be effective in treating various diseases such as diabetic nephropathy [5], [28] and cancer [12] in humans. We previously observed in Study 1 that OA ameliorated hyperglycemia beyond the cessation of its administration only in T2D mice, but not in T1D mice [13]. The same study also showed that the anti-hyperglycemic effect of OA was largely independent of its moderate effect on food intake during the period of OA administration. Indeed, the effect of triterpenoids in reducing hyperglycemia in T2D mice is consistent with a recent study in *db/db* mice using an OA analogue [5], [28]. The present study showed that the reduced hyperglycemia was maintained without any reduction in food intake during the period of post-OA treatment (Table 1). However, the molecular mechanisms underlying the sustained anti-hyperglycemic effects of OA were not investigated. The present study (Study 2) confirmed that the metabolic effect on hyperglycemia induced by OA in T2D is memorized after the period of the treatment. As our previous study revealed that the liver is a major target site [13], we next investigated the molecular mechanisms underlying this sustained anti-hyperglycemic effects (metabolic memory) with a focus on FoxO1, a master transcription factor regulating hepatic gluconeogenesis. The present study found that OA triggered a marked increase in the phosphorylation and acetylation of FoxO1 and these post-translational regulations were memorized, leading to the sustained inhibition of G6Pase expression (hence potentially reducing hepatic glucose production) well beyond the cessation of OA treatment. This mechanism is supported by a subsequent study showing the suppression of the increased hepatic glucose production from the gluconeogenesis with pyruvate as the substrate in HF-fed mice. These findings indicate, for the first time, a plausible mechanism of the metabolic memory for the therapeutic effect of a triterpenoid on hepatic glucose metabolism and glycemic control in a mouse model of T2D.

Liver is a major metabolic organ to maintain plasma glucose levels particularly during fasted states by gluconeogenesis or glycogenolysis. Excess hepatic glucose production is a major cause of hyperglycemia in T2D due to a diminished ability of insulin to suppress gluconeogenesis and/or glycogenolysis [29], namely hepatic insulin resistance. G6Pase is a rate-limiting enzyme controlling hepatic glucose production and this enzyme is largely regulated at the level of mRNA expression [30]. While we have observed a suppression of G6Pase in post-OA treatment in Study 1, whether G6Pase was already suppressed during OA treatment was not studied [13]. The present study showed that G6Pase expression was reduced during OA treatment along with the attenuation of hepatic steatosis. As a result, both the fasting hyperglycemia and pyruvate intolerance of OA-treated T2D mice were almost reduced to the normal level of CH-fed mice, suggesting the anti-hyperglycemic properties of OA are most likely due to the inhibition of hepatic glucose production. Moreover, the anti-hyperglycemic effect of OA observed in the present study is consistent with a recent study in *db/db* mice showing reduced hyperglycemia and hepatic G6Pase expression in response to OA treatment [11].

Hepatic gluconeogenesis is under the direct regulation of FoxO1, which mediates the expression of key genes of gluconeogenic pathway including G6Pase [31]. The transcriptional activity of FoxO1 is regulated by post-translational modifications which determine its subcellular location, molecular half-life, and/or DNA-binding activity [32]. Amongst these modifications, both phosphorylation and acetylation have been reported to dampen the transcriptional activity of FoxO1. Phosphorylation at serine 256 has been demonstrated to suppress FoxO1 transactivation by promoting its nuclear to cytosol shuttling [33]. Furthermore, acetylation at the various lysine residues has been found to attenuate the DNA binding activity of FoxO1 along with an increased sensitivity to the serine phosphorylation mediated by Akt [34], [35]. Indeed, we found for the first time that OA treatment triggered a marked and persistent acetylation of FoxO1 at lysine 259, 262 and 271 residues. The phosphorylation/acetylation of FoxO1 leads to its expulsion from the nucleus into the cytosol for ubiquitination-dependent proteasome degradation [36], this may explain the reduced expression of this protein in response to OA treatment.

Acetylation is controlled by HATs and HDACs [37], and this is crucial to the regulation of non-histone proteins, particularly FoxO1 [38]. Intriguingly, the increase in FoxO1 acetylation was sustained after the cessation of OA treatment and there was a matching increase in HAT1 and the serine phosphorylation of two specific Class IIa HDACs, namely HDAC4 and HDAC5. The phosphorylation of these HDACs potentially provides docking sites for the chaperone protein 14-3-3 which in turns promotes their nuclear export into the cytosolic compartments where they remain inactive [39], [40]. Additionally, HATs may play a direct role in regulating FoxO1 independent of HDACs. For example, increased HAT activity acetylates (thus represses) FoxO-mediated responses in C2C12 cells in response to dexamethasone and starvation [41], which are known to increase hepatic gluconeogenesis. The concomitant increased availability of HAT1 and decreased activity of Class IIa HDACs initiated by OA are likely to trigger a sustained shift in the equilibrium of FoxO1 modification towards enhanced protein acetylation status. Importantly, this shift in acetylation of FoxO1 is sustained beyond the period of OA treatment. Thus, our novel findings suggest acetylation may act in concert with phosphorylation to constitute a metabolic memory on FoxO1, repressing its transcriptional activity on gluconeogenic genes leading to the long-lasting glycemic control in the OA-treated mice.

One of the most intriguing novel observations in the present study is that the changes in Class IIa HDACs, HAT1, FoxO1 and G6Pase appeared to be memorized long after the direct action by OA *per se*. Although the sustained phosphorylation of FoxO1 at serine 256 strongly correlates with the increased activity of Akt as well as the persistent improvement of glycemic control, these changes are not due to the increased blood insulin level in both during and post-OA treatment. Class IIa HDACs play a regulatory role in physiological insulin action including the suppression of glucose uptake and glucose transporters expression in skeletal muscles [42], [43], and reduction of the acetylation of the insulin receptor substrate interfering with the proper insulin signal transduction in the liver [44]. This may explain the effects on glycemic control during OA treatment.

Activation of AMPK has been shown to induce the inhibitory acetylation of FoxO1 via phosphorylation of HDAC 4 and 5, and down-regulate G6Pase expression in the liver [14], [45]. Consistent with our previous reports [4], [10], the present study found that AMPK pathway was activated during OA administration. As AMPK can suppress lipid synthesis by inhibiting SREBP-1c [7], [10], which is a master transcriptional factor of lipogenic enzymes, this may explain the reduction in ACC, FAS and SCD-1 during OA administration. However, the sustained reduction in hyperglycemia after the cessation of OA treatment is clearly independent of the lipogenic pathway in the liver because the effects on SREBP-1c, ACC, FAS and SCD-1 have all subsided. These results also indicate that FoxO1 is unlikely to be a key regulator of lipogenic enzymes in the present study as previously suggested [46]. While activated AMPK can explain the suppression of hepatic gluconeogenesis by phosphorylating and acetylating FoxO1 as reported [23], [24], the sustained changes in HAT1 and Class IIa HADCs after the cessation of OA treatment do not require the simultaneous presence of chronic activation of AMPK. Thus far, we are not aware of similar report for other anti-diabetic agents which activate AMPK by different mechanisms. Further studies are needed to investigate whether the activation of AMPK is a prerequisite for the initiation of metabolic memory as reported for its effect on viral infection [21].

Apart from AMPK, Akt is another key regulator of FoxO1-induced suppression of hepatic gluconeogenesis [27]. Interestingly, the OA-induced increase in Akt phosphorylation is sustained 4 weeks after the cessation of OA treatment. This suggests that the sustained activation of Akt may also mediate the suppression of FoxO1 and G6Pase expression. It has been reported that the phosphorylation of Akt can be increased by chronic inhibition of HDACs [47], [48]. The inhibition of HDAC4 and 5 (increased phosphorylation) in the present study is consistent with this notion.

As recently reviewed [3], [12], OA widely presents in the plant kingdom such as olive products. It has been tested in humans for other conditions including the treatment for cancer [12] and hepatitis without serious toxicity reported [3]. An analogue of OA has also been shown to significantly improve diabetic nephropathy in humans [5], [28]. These reports suggest that OA may have a favorable safe profile in humans. While specific clinical trials are required to determine the safety of OA, our findings in this study provide a proof of principle for the potential use of OA or its analogues for the treatment of T2D.

**Figure 8:**
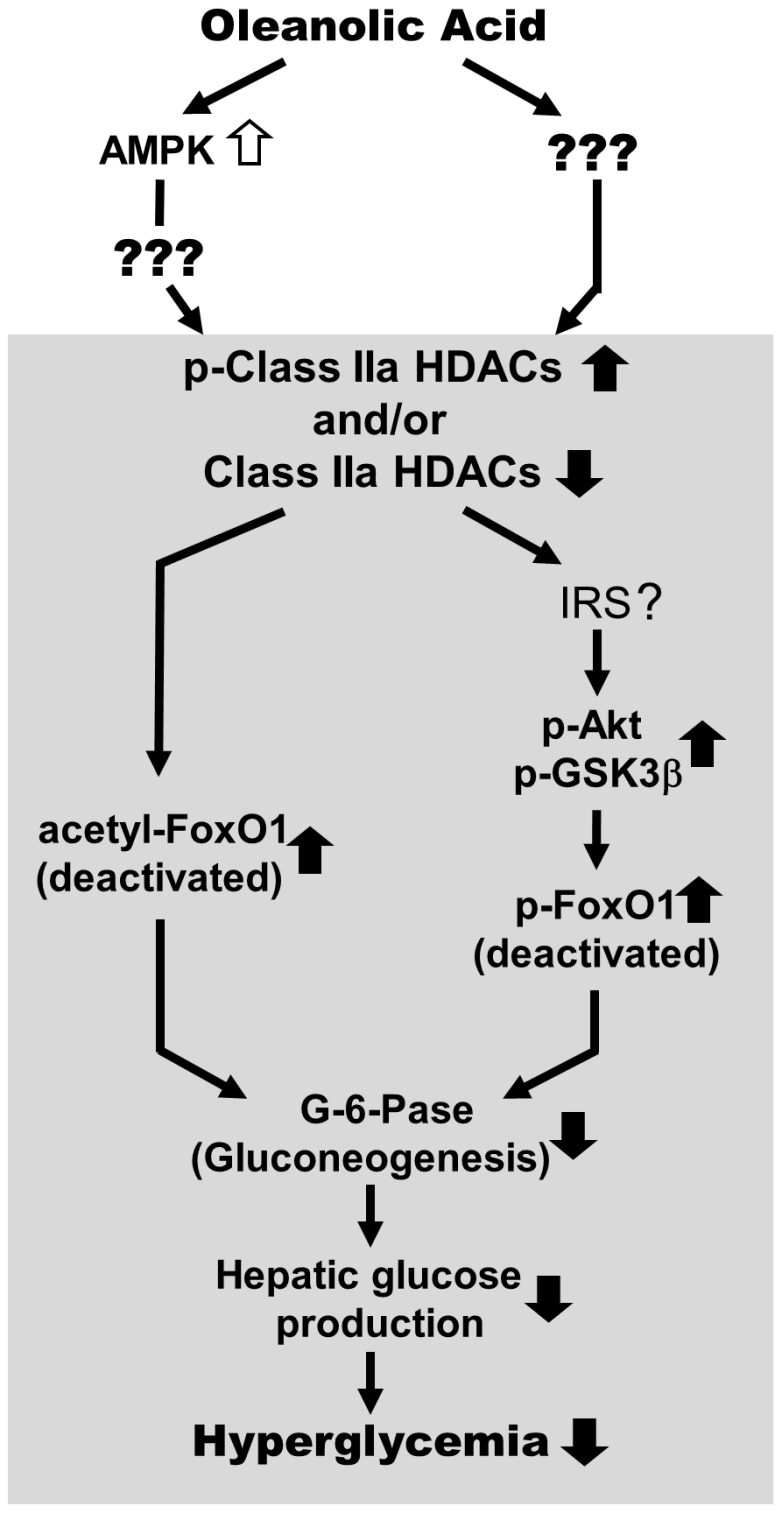
Proposed role of the hepatic HDACs/FoxO1 axis in the sustained reduction of hyperglycemia post-OA treatment. Administration of oleanolic acid stimulates AMPK activity which leads to an increased inhibitory phosphorylation of class IIa HDACs. The suppression of class IIa HDACs (and their possible reduction in the nucleus) induces acetylation and phosphorylation of FoxO1 as suggested [14]. The sustained inactivation of FoxO1 either by its acetylation and/or phosphorylation may contribute to the persistent anti-hyperglycemia effect of OA on HF-STZ induced T2D mice. The mechanism for the sustainability of these effects in the absence of AMPK activation (depicted in the shadow area) during the period of post-OA treatment is yet to be determined.

In summary, our findings of the memorized changes in Class IIa HDACs, acetylation and phosphorylation of FoxO1 in Study 2 provide novel insights into the mechanisms underlying the persistent anti-hyperglycemic effects observed post-OA treatment (Fig. 8). These modifications constitute a metabolic memory at the post-translational level leading to a suppression of hepatic gluconeogenesis via FoxO1 inhibition. Although the proposed mechanism requires further study, our results suggest a potential of pentacyclic triterpenoid class compounds as a long-lasting therapeutic approach for T2D. In a broader sense, our mechanistic data on OA also provide a basis for targeting Class IIa HDACs and FoxO1 in the gluconeogenic pathway for the sustained treatment of T2D.
